# Consecutive large dengue outbreaks in Taiwan in 2014–2015

**DOI:** 10.1038/emi.2016.124

**Published:** 2016-12-07

**Authors:** Sheng-Fan Wang, Ko Chang, El-Wui Loh, Wen-Hung Wang, Sung-Pin Tseng, Po-Liang Lu, Yen-Hsu Chen, Yi-Ming Arthur Chen

**Affiliations:** 1 Department of Medical Laboratory Science and Biotechnology, Kaohsiung Medical University, Kaohsiung 80708, Taiwan; 2 Center for Infectious Disease and Cancer Research, Kaohsiung Medical University, Kaohsiung 80708, Taiwan; 3 Department of Laboratory Medicine, Kaohsiung Medical University Hospital, Kaohsiung 80708, Taiwan; 4 Department of Medicine, Faculty of Medicine, College of Medicine, Kaohsiung Medical University, Kaohsiung 80708, Taiwan; 5 Division of Internal Medicine, Kaohsiung Municipal Hsiao-Kang Hospital, Kaohsiung Medical University, Kaohsiung 81267, Taiwan; 6 Center for Evidence-based Health Care, Taipei Medical University—Shuang Ho Hospital, New Taipei City 23561, Taiwan; 7 Division of Infection Disease, Department of Internal Medicine, Kaohsiung Medical University Hospital, Kaohsiung 80708, Taiwan; 8 Department of Microbiology and Immunology, College of Medicine, Kaohsiung Medical University, Kaohsiung 80708, Taiwan; 9 Center for Dengue Fever Control and Research, Kaohsiung Medical University, Kaohsiung 80708, Taiwan

**Dear Editor**,

The World Health Organization (WHO) has recently raised a warning that many countries have dengue epidemics or outbreaks. Major dengue fever (DF) cases were reported in Southeast Asia.^1^ DF is a vector-borne disease caused by dengue virus that belongs to the genus *Flavivirus* in the family Flaviviridae. Dengue viruses are geographically distributed by two competent vectors, *Aedes aegypti* and *Aedes albopictus*. The *Aedes aegypti* mosquitoes are predominant in southern Taiwan and highly correlated with dengue epidemics or outbreaks.^2^ Previous reports have indicated that dengue epidemics or outbreaks have mainly occurred in the south of Taiwan, such as Tainan and Kaohsiung,^3^ which are neighboring cities. Dengue activity has mainly been found in Kaohsiung city.^3^ The co-circulation of two or even four dengue serotypes (DENV-1–4) has been reported in Kaohsiung city.^4^

A severe DF outbreak occurred in Taiwan in 2014. A total of 15 732 DENV laboratory confirmed cases were reported by the Centers for Disease Control, Taiwan (Taiwan CDC)^4^ (Supplementary Figure S1), including 15 492 indigenous and 240 imported cases (Supplementary Table S1). A total of 15 043 cases (96%) were reported in Kaohsiung city.^4^ We previously reported a correlation between the underground pipeline leaking gas explosion event, which was followed by continuous heavy rains, with the 2014 DF outbreak in Kaohsiung (*P*<0.0001; *r*=0.87).^5^ The cavities created by the explosion might have led to an increase in stagnant waters suitable for breeding of the *Aedes* mosquitoes. We found that the Breteau index for measuring the density of mosquitoes increased from 10%–19% to 35%–49% from July to September in Kaohsiung. Serotyping using real-time reverse transcription polymerase chain reaction (real-time RT-PCR) for the *NS5* gene and detection with type-specific primers and probes^6^ indicated DENV-1 as the predominant strain (Figure 1A; Supplementary Table S1). This 2014 DF outbreak was initiated by imported DF cases, and their viral envelope sequences were similar to the 2013 Indonesia isolates (Figure 1A; Supplementary Table S1).

However, a consecutive larger DF outbreak occurred in Taiwan in 2015, and a total of 43 784 DF cases were reported by the Taiwan CDC^4^ (Supplementary Figure S1). Among them, 362 were imported cases (Supplementary Table S1). Most of the DF cases were distributed in Tainan (22 777; 52%) and Kaohsiung (19 784; 45%).^4^ Serotyping data indicated DENV-2 as the predominant strain in the 2015 DF outbreak (Figure 1A; Supplementary Table S1). DENV-2 infected cases were reported initially in May, and then the number surged to its highest peak in September but gradually decreased after October in Tainan city. In addition, the Taiwan CDC reported that DENV-2 was disseminated to the neighboring city, Kaohsiung, after July.^4^ We collaborated with the Kaohsiung Medical University Hospital (KMUH) and obtained 2000 DENV-positive serum samples in which the subjects were infected in 2015 as confirmed by the laboratory of Taiwan CDC.^4^ Written informed consent was obtained from each participant. All study protocols were approved by the institutional review board of the Kaohsiung Medical University. The serotype of 940 samples was determined using virus isolation and real-time RT-PCR.^6, 7^ Most cases identified before July were DENV-1 infections. From August to September, 20% were DENV-1, and 80% were DENV-2. Samples collected after October were all DENV-2.

Phylogenetic analysis (a neighbor-joining tree with *p*-distance inferred with 1000 bootstrap replicates in MEGA program version 5 for a 1485-nt fragment spanning the full gene of the envelope glycoprotein)^8^ proved that the DENV-2 isolated in Kaohsiung after August clustered with the 2015 outbreak-associated DENV-2 Tainan strain (bootstrap value was 100). However, it did not cluster with previous Taiwanese epidemic DENV-2 isolates (Figure 1A, left). Notably, the Taiwanese 2015 DENV-2 outbreak strains phylogenetically clustered with the 2014 China and 2015 Indonesia isolates. We suggest that this DF outbreak may have been caused by the imported DENV-2 infected cases from China or Indonesia. A similar phenomenon was found in the 2014 DENV-1 outbreak in Kaohsiung, which may have been caused by the imported cases infected with the 2013 Indonesia strain (Figure 1B, right) (Supplementary Table S1). We also found that the 2014 DENV-1 outbreak-associated strains persisted in Kaohsiung until September of 2015. After October, they switched to DENV-2 strains that originated from Tainan. Previous reports indicate that Taiwan is not a dengue endemic region but that the constant importation of DENVs from neighboring Southeast Asian countries is the major cause of dengue epidemics or outbreaks.^9^ Similar results were found in this study (Supplementary Table S1).

A higher DF death rate was observed in the 2015 outbreak (5.15**‰**), which was more than three times higher than the 2014 outbreak (1.65**‰)** (Supplementary Table S2). There were 136 and 674 dengue hemorrhagic fever or dengue shock syndrome (DHF/DSS) cases in 2014 and 2015, respectively. The DHF/DSS death rates were 19.1% (26/136 cases) and 34.6% (224/674 cases) in 2014 and 2015, respectively (Supplementary Table S2). The death rate in 2015 DHF/DSS cases was higher than previous dengue epidemics or outbreaks. Several studies revealed that DHF/DSS occurred in individuals with secondary heterotypic DENV infections and some in primary infections in infants born to dengue-immune mothers.^10, 11^ Pre-existing antibodies may cross-react and enhance viral entry into host cells through the antibody-dependent enhancement effect in heterotypic secondary DENV infections.^10, 11^ However, the mechanism is still not fully understood. It is possible that some DF cases might have previously been infected with the 2014 DENV-1 or other types of DENV and were subsequently infected with the 2015 DENV-2 outbreak strains, which resulted in severe dengue syndromes. In addition to antibody responses, pro-inflammatory cytokine up-regulation, and T-cell activation, certain vasoactive factors may also increase vascular permeability and lead to DHF/DSS.^10, 11^ The different serotypes and genotypes of DENVs have been reported to affect clinical outcomes of dengue infection.^10^ Climate change is one of the most important environmental factors that correlates with disease occurrence.^12^ Climate is known to affect DENV and vector populations directly and indirectly,^13^ and temperature influences vector development rates, mortality, and behavior as well as control of viral replication within the mosquito.^13^ We examined the relationship of climate change with 2014–2015 DF outbreaks. The mean temperatures in 2014 and 2015 were 23.6 and 25.2 °C, respectively, which was 0.3–2 °C higher than that in the past 10 years. Similarly, temperature changes were significantly correlated with DF outbreaks in 2014 and 2015 (*P*=0.01, *r*=0.76; *P*=0.008, *r*=0.82, respectively).

We also analyzed the potential role of rainfall and consecutive rainy days with DF outbreaks. The results indicated that there was a significant correlation between the number of 2014–2015 DF cases and precipitation (*P*=0.006, *r*=0.79) and consecutive days with rain (*P*=0.002, *r*=0.67). The DF cases significantly increased 1–2 months after the long consecutive days of rain in Tainan and Kaohsiung (*P*<0.01). The correlation might be caused by the long-term wet environment that is suitable for egg hatching and larva survival of the mosquitoes. Recent reports have proved that precipitation influences habitat availability for the *Aedes* mosquito and its pupae. In addition, rainfall and humidity influenced land cover and land use, which could promote or impede the growth of vector populations.^14, 15^

Recently, dengue outbreaks and epidemics have been reported in several countries. We suggest that dengue may be an emerging or re-emerging problem that draws our attention.

## Figures and Tables

**Figure 1:**
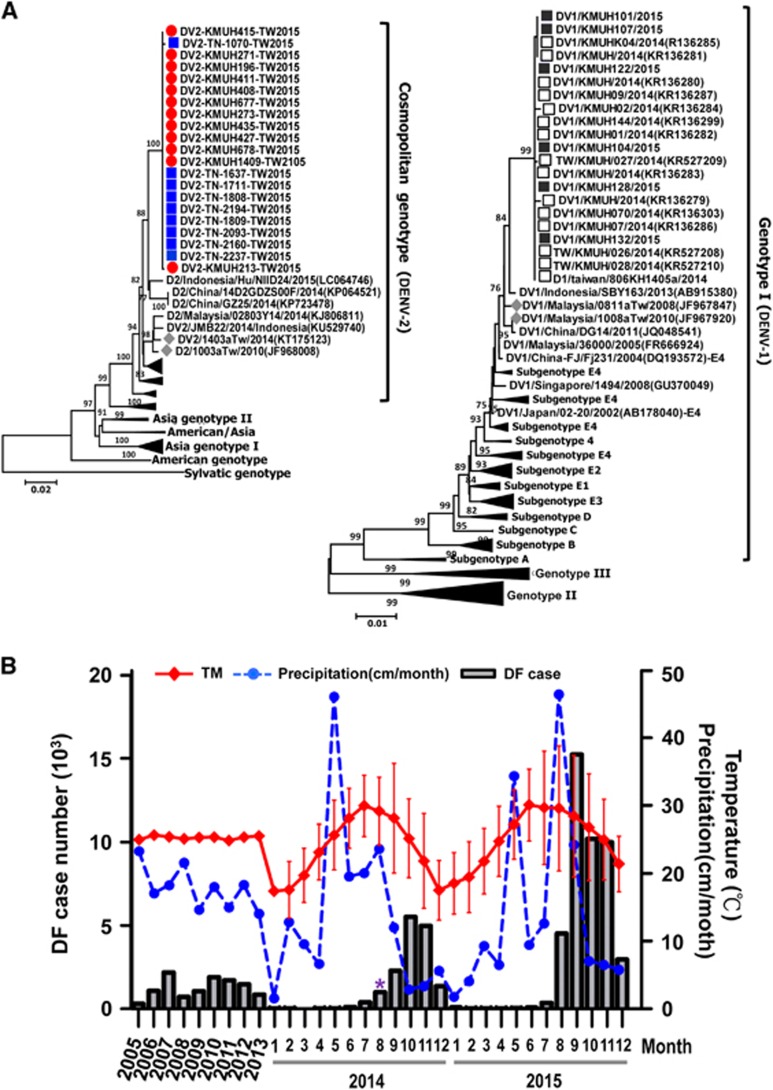
Dengue fever (DF) outbreaks in Taiwan during 2014–2015. (**A**) Phylogenetic tree analyses of Taiwanese 2014 (the right) and 2015 (the left) dengue serotype 1 and 2 outbreak-associated isolates. The nucleotide sequences of complete *E*-genes of DENV strains were aligned, edited and analyzed using ClustalW software (http://bioedit.software.informer.com/7.2/). The phylogenetic analysis was performed using MEGA version 5. Consensus neighbor-joining trees were obtained from 1000 bootstrap replicates.^8^ The red-filled circles and blue-filled squares indicate DENV-2 isolated from Kaohsiung and Tainan in the 2015 severe DF outbreak. The black-outlined and black-filled squares indicate the 2014 outbreak-associated DENV-1 isolates and 2015 DENV-1 epidemic strains in Kaohsiung city. The gray-filled diamonds in both phylogenetic trees indicate previous Taiwanese DENV epidemic strains. (**B**) The accumulative dengue infection cases in the past decade (left) and reported cases monthly in 2014–2015 (right) in Taiwan are shown. The association of dengue case numbers with temperature and precipitation is also illustrated. The asterisk (*) indicates the time of the gas explosion in Kaohsiung city.
