# Supplementary material for: Consecutive large dengue outbreaks in Taiwan in 2014–2015
Source: Emerg Microbes Infect. 2016 Dec 7;5(12):e123–. doi: 10.1038/emi.2016.124 (PMC5180368; doi:10.1038/emi.2016.124)
Supplement: Supplementary Figure S1 [file emi2016124x1.doc]

**
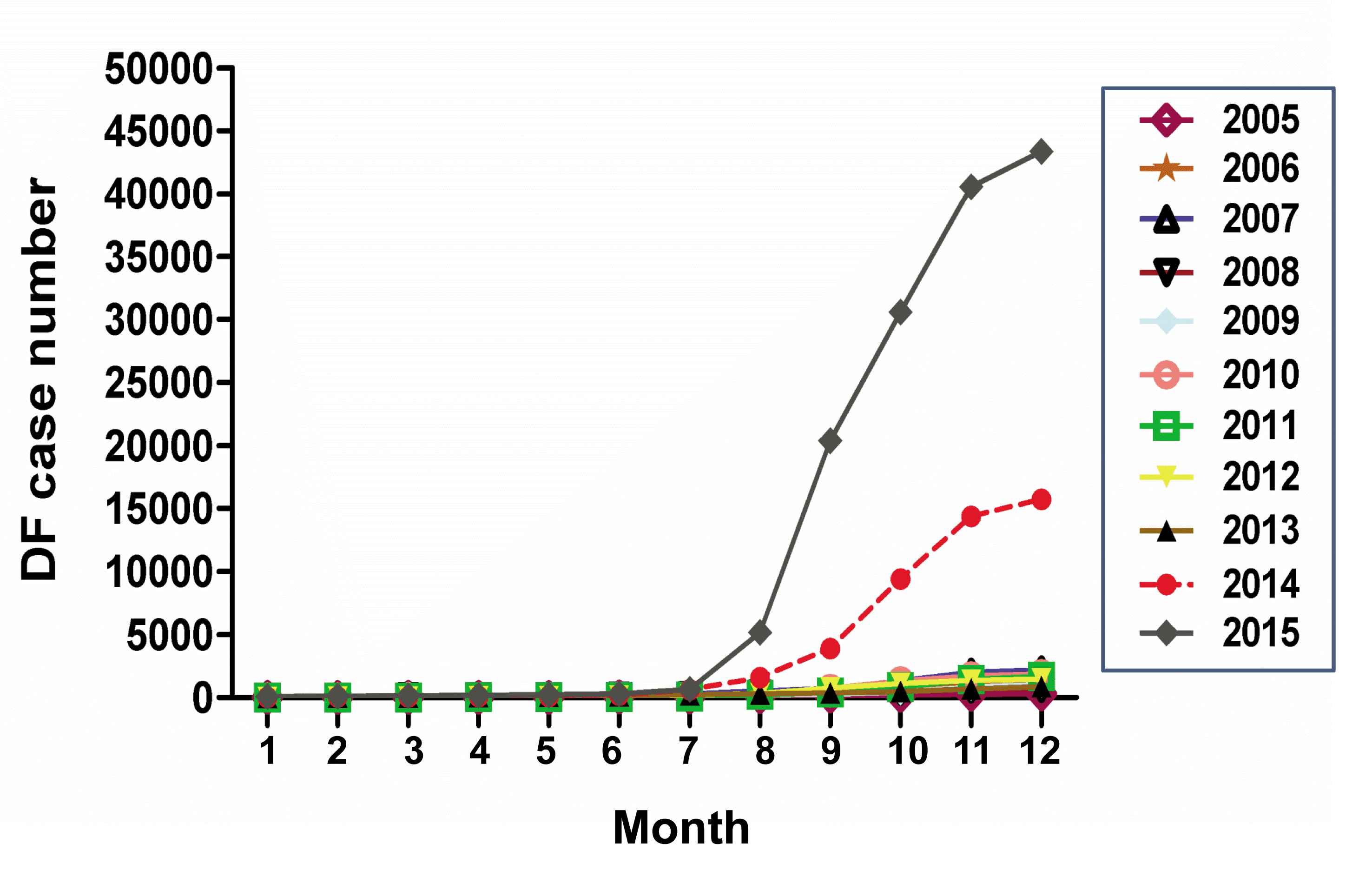
**

**Supplementary Figure S1** Accumulated dengue fever (DF) cases reported in Taiwan during 2005-2015. Accumulated number of DF cases reported annually in Taiwan during 2005-2015. The data was collected from Taiwan CDC. All the DF cases were laboratory confirmed.
