# Supplementary material for: Consecutive large dengue outbreaks in Taiwan in 2014–2015
Source: Emerg Microbes Infect. 2016 Dec 7;5(12):e123–. doi: 10.1038/emi.2016.124 (PMC5180368; doi:10.1038/emi.2016.124)
Supplement: Supplementary Table S1 [file emi2016124x2.doc]

**Supplementary Table S1 Dengue fever in Taiwan during 2000-2015**.

| **Year** | **Imported cases** |  | **Epidemic DENVs** | | **Phylogenetic related source** |
| --- | --- | --- | --- | --- | --- |
|  | **Imported Indigenous** |  | **Serotype** | **Genotype** |  |
| 2000 | 26 113 |  | DENV-4* | Genotype II | Thailand |
| 2001 | 54 227 |  | DENV-2* | Cosmopolitan | Philippines |
| 2002 | 52 5336 |  | DENV-1 | Genotype II | Indonesia |
|  |  |  | DENV-2* | Cosmopolitan | Philippines |
| 2003 | 59 86 |  | DENV-2* | Cosmopolitan | Philippines |
| 2004 | 91 336 |  | DENV-1* | Genotype II | Philippines |
|  |  |  | DENV-4 | Genotype II | Vietnam |
| 2005 | 104 202 |  | DENV-2 | Asian/America | Vietnam |
|  |  |  | DENV-3* | Genotype I | Philippines |
|  |  |  | DENV-3 | Genotype II | Vietnam |
| 2006 | 109 956 |  | DENV-2 | Asian I | Vietnam |
|  |  |  | DENV-3* | Genotype II | Cambodia |
| 2007 | 176 2000 |  | DENV-1* | Genotype I | Thailand |
|  |  |  | DENV-2 | Asian I | Vietnam |
| 2008 | 226 448 |  | DENV-1* | Genotype I | Vietnam/ Thailand |
|  |  |  | DENV-2 | Asian I | Cambodia |
| 2009 | 205 857 |  | DENV-1 | Genotype I | Thailand |
|  |  |  | DENV-2 | Asian I | Vietnam |
|  |  |  | DENV-3* | Genotype I | Philippines |
| 2010 | 303 1585 |  | DENV-1 | Genotype I | Vietnam/ Cambodia |
|  |  |  | DENV-2 | Cosmopolitan | Philippines |
|  |  |  | DENV-3* | Genotype I | Philippines |
|  |  |  | DENV-4 | Genotype II | Indonesia |
| 2011 | 157 1543 |  | DENV-1 | Genotype I | Dominican |
|  |  |  | DENV-2* | Cosmopolitan | Philippines |
|  |  |  | DENV-3* | Genotype I | Indonesia |
| 2012 | 207 1270 |  | DENV-1 | Genotype I | Dominican |
|  |  |  | DENV-2* | Cosmopolitan | Indonesia/Thailand |
|  |  |  | DENV-3 | Genotype I | Indonesia |
|  |  |  | DENV-4 | Genotype II | Philippines |
| 2013 | 261 596 |  | DENV-1 | Genotype I | Dominican /Malaysia |
|  |  |  | DENV-2* | Cosmopolitan | Indonesia |
|  |  |  | DENV-3 | Genotype I | Indonesia |
|  |  |  | DENV-4 | Genotype II | Philippines |
| 2014 | 245 15509 |  | DENV-1* | Genotype I | Indonesia |
| 2015 | 365 43419 |  | DENV-1 | Genotype I | Indonesia |
|  |  |  | DENV-2* | Cosmopolitan | Indonesia/China |

Footnote: The imported cases of dengue fever are defined as (a) coming from dengue endemic or epidemic regions or countries outside of Taiwan with a history of being bitten by mosquito within 15 days before the onset of illness and has no history of being bitten by mosquitoes in domestic regions, (b) the viral gene sequence (such as Envelope (*E*) gene) from the isolated virus is highly homologous with gene sequence that has been reported by the dengue endemic or epidemic countries from where the patients had travelled. An indigenous case is defined according to the absence of evidence for the case being imported. The data is adapted from the publications of Taiwan CDC^7,9^ and dengue public data resource (http://www.cdc.gov.tw /professional/index.aspx). The partial data of DENV serotyping and phylogeny were confirmed in our laboratory by using virus culture combined with real-time RT-PCR^6^, as well as phylogenetic analysis using *E* gene sequence^8^. “*” indicates the dominant strain in that year. All these DENVs were laboratory confirmed.
