# Supplementary material for: Consecutive large dengue outbreaks in Taiwan in 2014–2015
Source: Emerg Microbes Infect. 2016 Dec 7;5(12):e123–. doi: 10.1038/emi.2016.124 (PMC5180368; doi:10.1038/emi.2016.124)
Supplement: Supplementary Table S2 [file emi2016124x3.doc]

**Supplementary Table S**2 The incidence of DF and DHF/DSS occurrence in Taiwan during 2005-2015

| **YYear** | **2005** | **2006** | **2007** | **2008** | **2009** | **2010** | **2011** | **2012** | **2013** | **2014** | **2015** |
| --- | --- | --- | --- | --- | --- | --- | --- | --- | --- | --- | --- |
| **DF** | 306 | 1074 | 2179 | 714 | 1052 | 1896 | 1702 | 1478 | 860 | 15732 | 43522 |
| **DHF/DSS** | 3 | 19 | 12 | 5 | 11 | 21 | 22 | 36 | 16 | 136 | 647 |
| **Death case** | 0 | 4 | 1 | 4 | 4 | 2 | 5 | 7 | 0 | 26 | 224 |
| **DF Death rate( ‰)** | 0 | 3.73 | 0.45 | 5.61 | 3.81 | 1.05 | 2.94 | 4.74 | 0 | 1.65 | 5.15 |
| **DHF/DSS Incidence (%)** | 0.98 | 1.77 | 0.55 | 0.71 | 1.05 | 1.11 | 1.29 | 2.44 | 1.86 | 0.86 | 1.49 |
| **DHF/DSS death rate (%)** | 0 | 21 | 8.3 | 80 | 36.4 | 9.5 | 22.7 | 19.4 | 0 | 19.1 | 34.6 |

Footnote: dengue fever, DF; dengue hemorrhagic fever, DHF; dengue shock syndrome, DSS.
